# Research trends and hotspots in the relationship between outdoor activities and myopia: A bibliometric analysis based on the web of science database from 2006 to 2021

**DOI:** 10.3389/fpubh.2022.1047116

**Published:** 2022-10-26

**Authors:** Jingfeng Mu, Haoxi Zhong, Dan Zeng, Jingjie Fan, Mingjie Jiang, Meizhou Liu, Xinyi Shuai, Yanjie Chen, Shaochong Zhang

**Affiliations:** ^1^Shenzhen Eye Hospital, Jinan University, Shenzhen Eye Institute, Shenzhen, China; ^2^Affiliated Shenzhen Maternity & Child Healthcare Hospital, Southern Medical University, Shenzhen, China

**Keywords:** outdoor activities, myopia, bibliometrics, data visualization, CiteSpace

## Abstract

**Objectives:**

This study aimed to explore the current status, hotspots, and emerging research trends regarding the relationship between outdoor activities and myopia.

**Methods:**

Publications on the relationship between outdoor activities and myopia from 2006 to 2021 were collected from the Web of Science Core Collection database. CiteSpace (version 6.1.R2) was used to performed a bibliometric analysis, and R software (version 4.1.0) was used to visualize the trends and hot map of publications.

**Results:**

A total of 640 publications were collected and analyzed in the present study. China was the major contributor (*n* = 204), followed by the United States of America (*n* = 181) and Australia (*n* = 137). The United States of America had the most extensive foreign cooperation (centrality = 0.25), followed by Australia (centrality = 0.20). The National University of Singapore contributed the largest number of publications (*n* = 48), followed by Sun Yat-Sen University (*n* = 41) and the Australian National University (*n* = 41). Among institutions, Cardiff University in the United Kingdom had the most extensive foreign cooperation (centrality = 0.12), followed by the National University of Singapore (centrality = 0.11). Saw S from Singapore had the largest number of publications (*n* = 39), followed by Morgan I from Australia (*n* = 27) and Jonas J from Germany (*n* = 23). *Investigative ophthalmology* & *visual science* is the most important journal to study the relationship between outdoor activities and myopia. “Global Prevalence of Myopia and High Myopia and Temporal Trends from 2000 through 2050” published by Holden BA was the most cited paper in this field with 177 citations. Co-occurrence and burst analyses of keywords showed that research trends and hotspots in this field focused mainly on “risk,” “prevention” and “school”.

**Conclusions:**

The influence of outdoor activities on myopia remains a concern. In the future, deeper cooperation between countries or institutions is required to explore the effects of outdoor activities on myopia. Outdoor activities for the prevention of myopia and reduction of the risk of myopia among school students may be the focus of future research.

## Introduction

Myopia, a major public health concern, has become the leading cause of visual impairment worldwide. The global prevalence of myopia is 28.3%, and the prevalence of myopia in Asia is significantly higher than that in other regions (1). Approximately 80–90% of high school students in China suffer from myopia, with high myopia being the most common sub-type at 10–20% (2). Studies have predicted that by the year 2050, nearly half of the world's population would have contracted myopia, of which nearly one-tenth will be high myopia (3, 4). Myopia, especially high myopia, may result in a variety of complications, including cataracts, retinal detachment, glaucoma, macular holes, and even blindness (5–7).

Lack of outdoor activities has been reported as the major risk factor for myopia in children and adolescents (8). Studies have reported that the prevalence of myopia in these age groups can be reduced by extending the duration of their outdoor activities (9, 10). According to a meta-analysis, outdoor light exposure can reduce the incidence of myopia, slow its progression, and reduce axial elongation (11). The mechanisms by which outdoor activities affect the occurrence and development of myopia remain unclear. However, it has been postulated that factors such as light exposure, release of dopamine along with vitamin D, circadian rhythms, and near work could be possible explanations (12–15).

Bibliometrics is an important method to discover the law of development of discipline. At present, bibliometrics has been widely used in many fields, such as economics (16), environmental science (17), information management (18), social sciences (19) and biomedicine (20, 21). The number of published research articles on myopia has increased worldwide. Some researchers have applied bibliometrics to the field of myopia in recent years, such as trends in research related to high myopia (22), myopia genetics (23), myopia management (24) and publications on myopia (25).

To the best of our knowledge, no bibliometric analysis has discussed the relationship between outdoor activities and myopia. Thus, the present study was undertaken to explore the current status, hotspots, and emerging trends of research regarding the relationship between outdoor activities and myopia, so as to understand the current research status and future development trend in this field. Publications on the relationship between outdoor activities and myopia from 2006 to 2021 were collected from the Web of Science Core Collection database. CiteSpace (version 6.1.R2) was used to performed a bibliometric analysis, and R (version 4.1.0) was used to visualize the trends and hot map of publications.

## Materials and methods

### Literature resources

We used the Web of Science Core Collection database, which contains more than 12,000 high-impact academic journals, for document retrieval (26, 27). The literature search strategies were “(TS = (myopia and physical activity^*^)) OR (TS = (myopia and outdoor^*^)) OR (TS = (refractive error^*^ and physical activity^*^)) OR (TS = (refractive error^*^ and outdoor^*^)).” We included original research articles and reviews published in English language between January 1, 2006, and December 31, 2021. The literature search was completed on October 9, 2022. We excluded repetitive literature, meeting abstracts, news items, editorial materials, letters, books, early access and proceedings papers.

### Data extraction and analysis

Two researchers independently conducted the literature search. Any controversial data obtained was discussed by the researchers, and a consensus was reached. CiteSpace software is a Java-based application program developed by Professor Chaomei Chen, which visualizes the interrelationship between literature based on co-citation networks (28). In this study, CiteSpace (version 6.1. R2) was used to map the scientific knowledge, and R software (version 4.1.0) was used to visualize the trends and hot map of published research articles. The countries or regions, institutions, journals, and authors of the literature were analyzed to discern the structure of knowledge in this field. Co-citation networks, keywords, and words with strong citation bursts were analyzed to recognize the hotspots, frontiers, and research trends. The network of scientific knowledge consists of nodes and links between nodes. In different networks of scientific knowledge, nodes represent keywords, authors, and so on. The size of the node represents the frequency of publication or citation. The color of the node represents the year, and a warmer color indicates a more recent year. Nodes with centrality greater than 0.1 are often seen as key points. The links between nodes represent co-citation or collaboration or co-occurrence. *Q* value and *S* value are provided in CiteSpace, which are evaluation indexes of mapping effect of the network. *Q* value is greater than 0.3 indicates that the community structure of the network of scientific knowledge is significant, and *S* value is greater than 0.5 indicates that the clustering of the network of scientific knowledge is reasonable (29).

## Results

### Publication and citation trends

**Figure 1:**
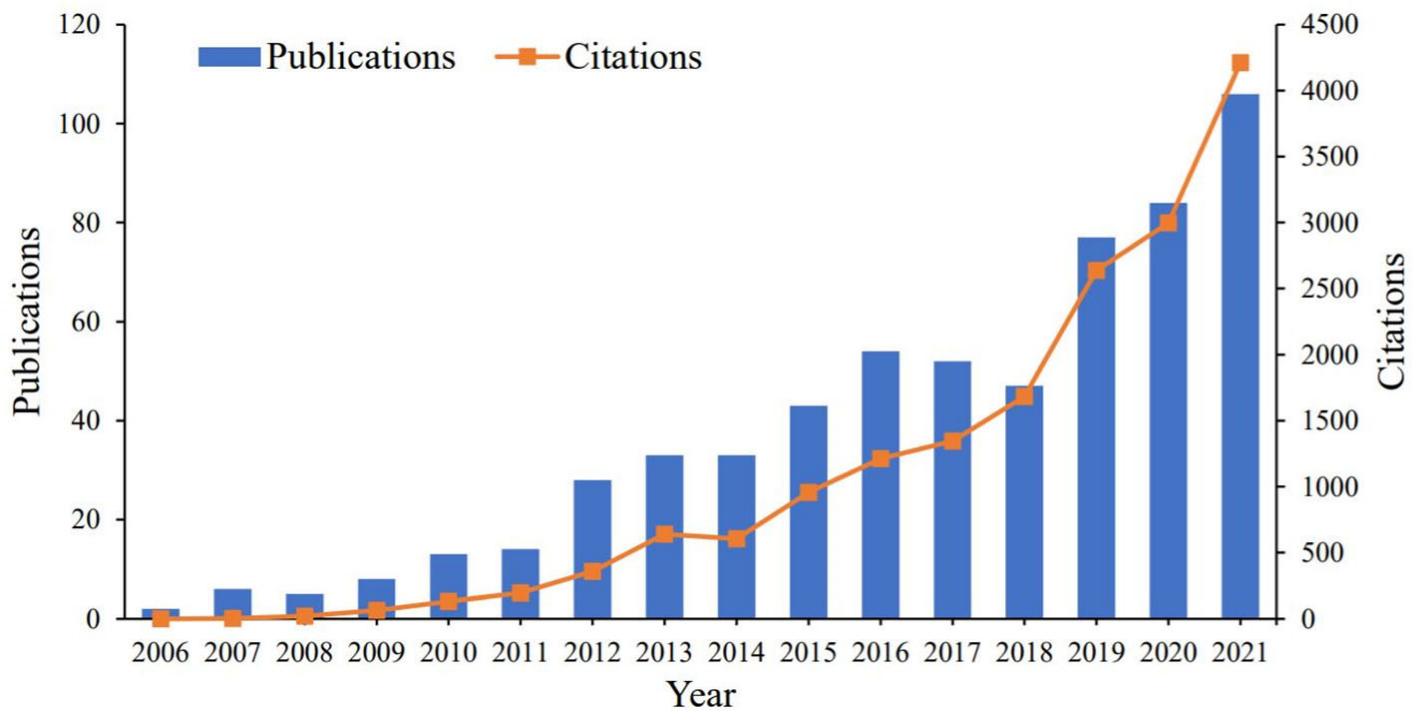
Trends of publications and citations about studies on the relationship between outdoor activities and myopia from 2006 to 2021.

A total of 640 published research articles (567 original research articles and 73 reviews) between January 1, 2006, and December 31, 2021 were included in this study. The annual number of publications between 2006 and 2009 was less than ten, and the annual number of citations was less than 100. The number of publications and citations has increased annually since 2010, reaching 106 and 4,211, respectively, in 2021 (Figure 1). The publications were cited 20,846 times (13,261 times after removing self-citations) between 2006 and 2021. The average number of citations and h-index per paper were 32.57 and 72.0, respectively.

### Analysis of countries/regions

**Figure 2:**
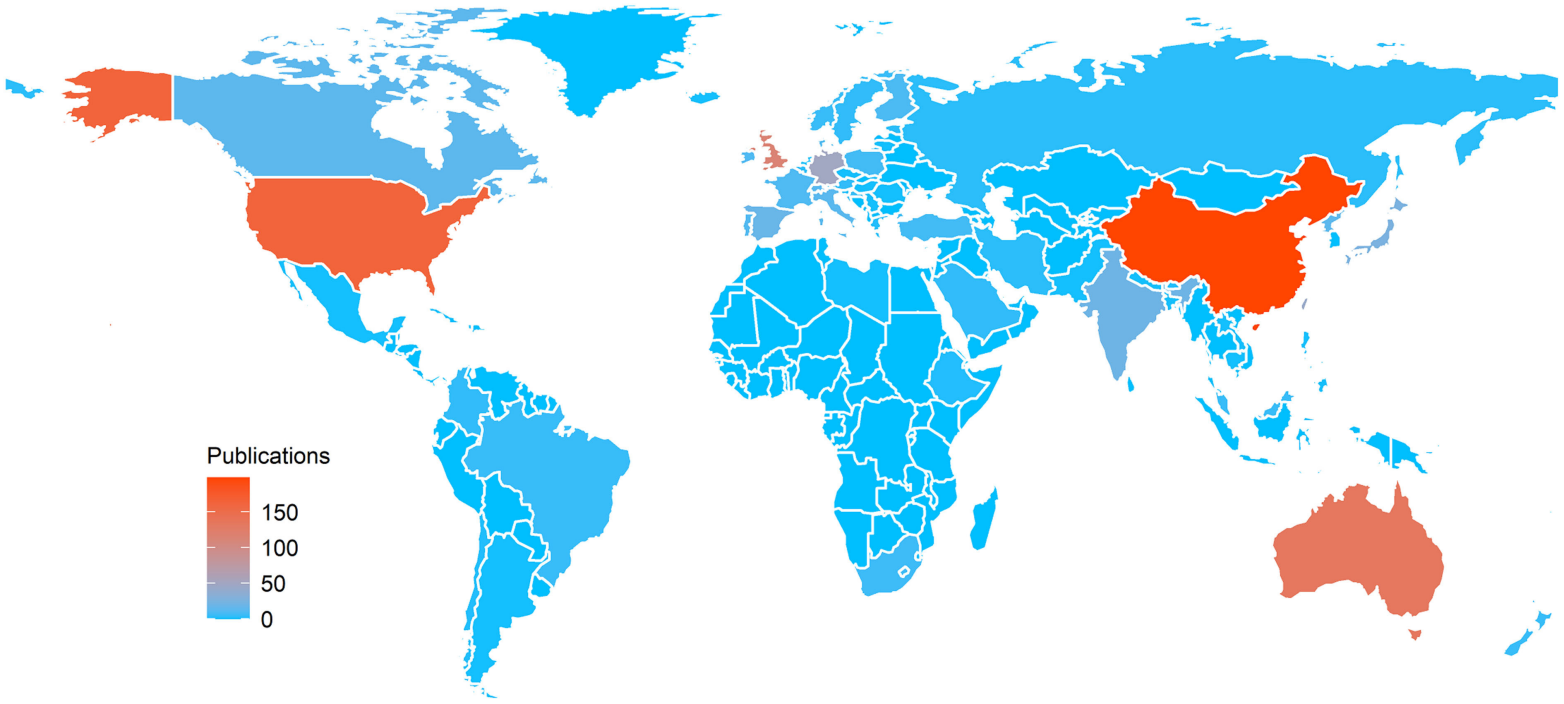
Hot map based on publications according to countries/regions.

**Figure 3:**
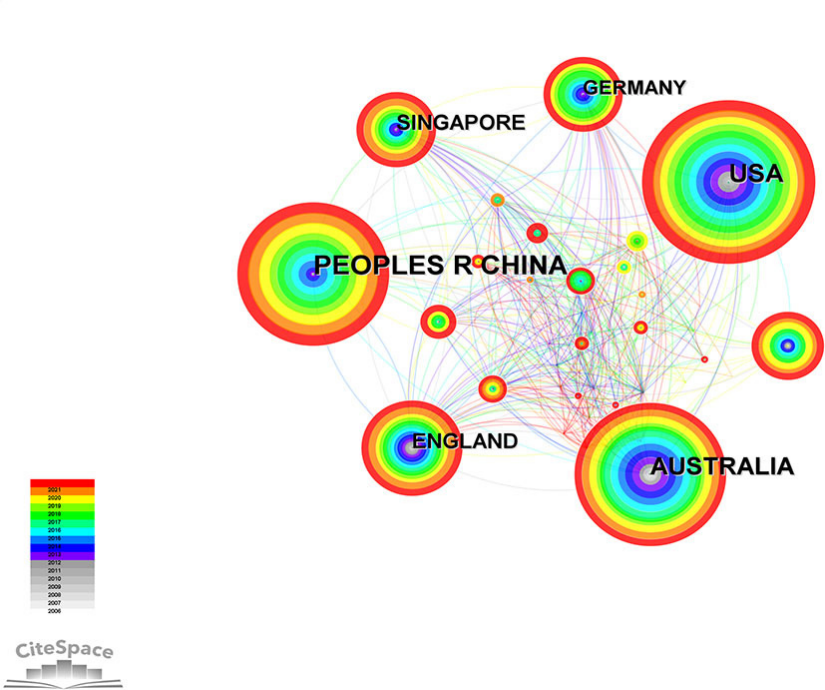
Countries of studies on the relationship between outdoor activities and myopia from 2006 to 2021. USA, United States of America.

**Figure 4:**
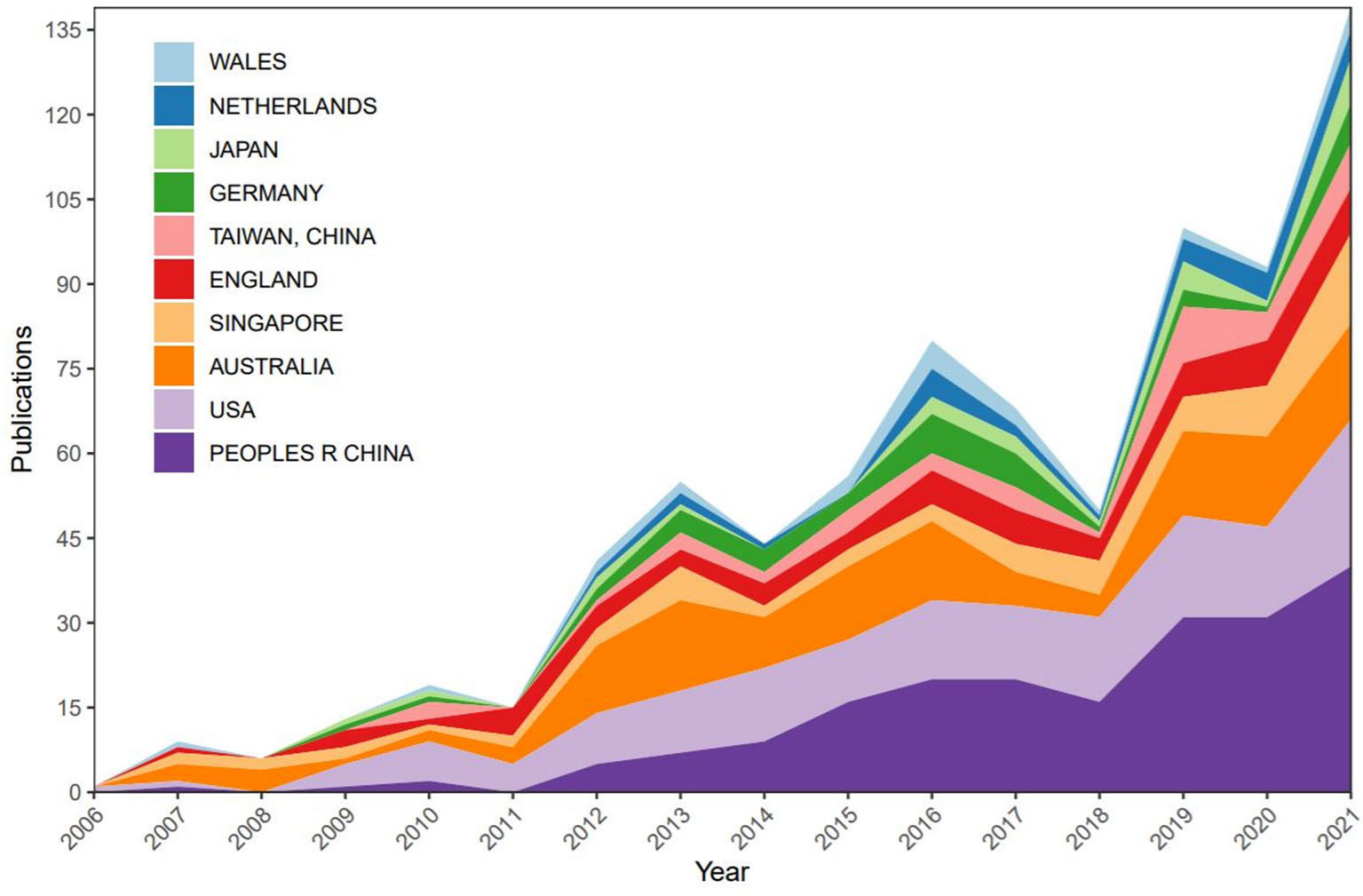
Trends of publications about studies on the relationship between outdoor activities and myopia from 2006 to 2021 in the top 10 countries. USA, United States of America.

Sixty two countries/regions published papers related to this field (Figure 2). The network diagram of papers published in these countries/regions was constructed using CiteSpace, and it comprised 62 nodes and 395 links (Figure 3). The *Q* value and *S* value of the network was 0.6177 and 0.8583, respectively. China had the largest number of publications (*n* = 204), accounting for 31.9% of all publications. The United States of America (*n* = 181) and Australia (*n* = 137) ranked second and third, respectively. The United States of America had the highest centrality (centrality = 0.25) and extensively cooperated with other countries, followed by Australia (centrality = 0.20) and England (centrality = 0.15) (Table 1). Analysis of the top 10 countries/regions with the maximum number of publications revealed that China was relatively late in conducting related research, and their number of publications did not increase until 2012 (Figure 4).

**Table 1:** Top 10 countries/regions with the most publications from 2006 to 2021.

**Country/regions**	**Counts**	**Total citations**	**Average citation per paper**	**H-index**	**Centrality**
PEOPLES R CHINA	204	5,802	28.30	40	0.01
USA	181	6,318	34.91	43	0.25
AUSTRALIA	137	7,876	57.07	48	0.20
SINGAPORE	68	3,400	50.00	29	0.08
ENGLAND	65	3,597	56.20	31	0.15
TAIWAN, CHINA	44	1,821	41.39	20	0.01
GERMANY	43	1,846	43.95	23	0.04
JAPAN	27	821	30.41	16	0.03
NETHERLANDS	26	850	32.69	15	0.07
WALES	25	1,082	43.28	18	0.04

USA, United States of America.

### Analysis of institutions

**Figure 5:**
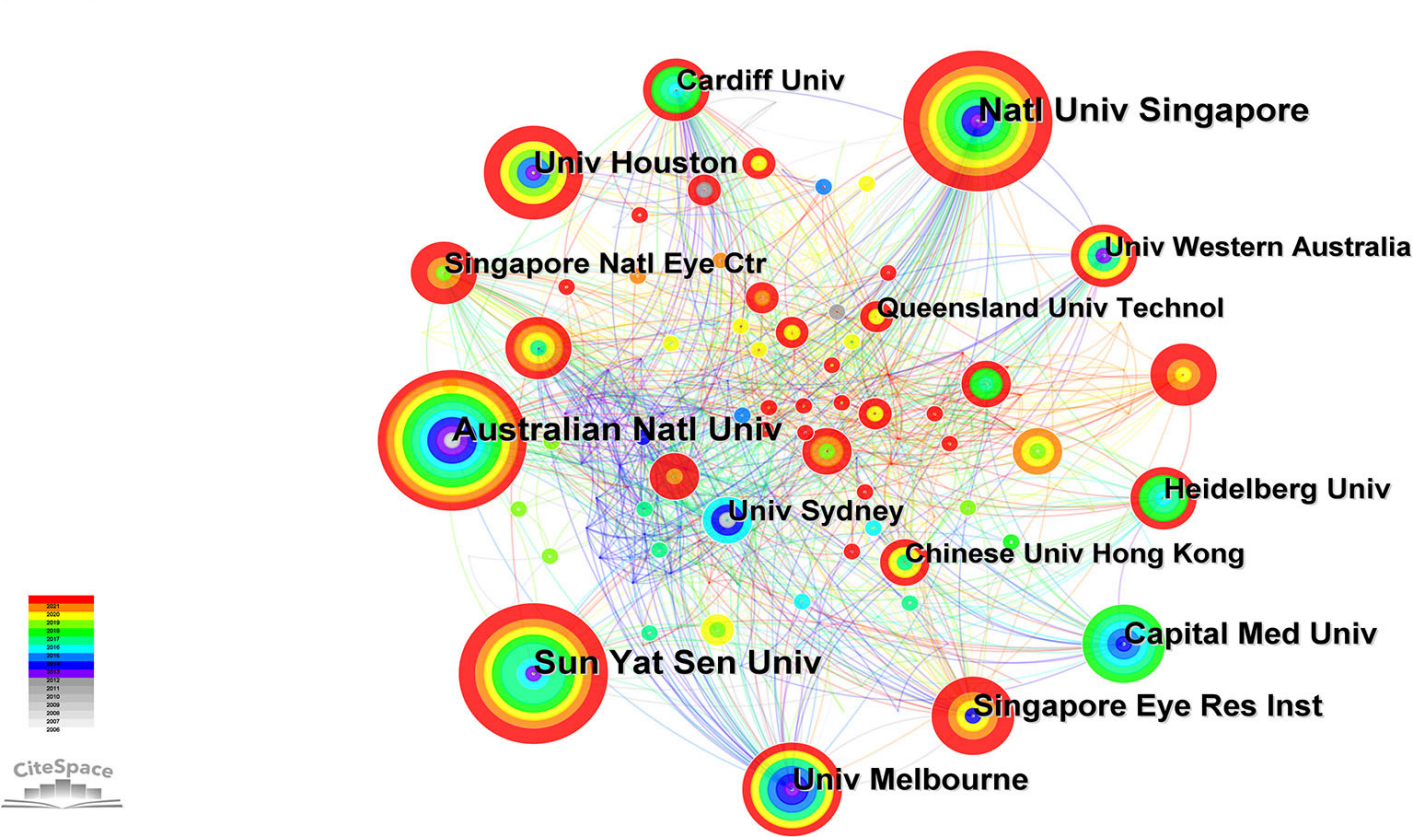
Institutions of studies on the relationship between outdoor activities and myopia from 2006 to 2021.

A total of 301 institutions published papers related to this field. The network diagram of these institutions published papers was constructed using CiteSpace, and it comprised 301 nodes and 1,178 links (Figure 5). The *Q* value and *S* value of the network was 0.6177 and 0.8583, respectively. The diagram showed extensive cooperation among institutions. The National University of Singapore had the largest number of publications (*n* = 48), followed by Sun Yat-Sen University (*n* = 41) and the Australian National University (*n* = 41). Among the top 10 institutions with the majority of publications, three were from Singapore, two each from China and Australia, and one each from the United States of America, the United Kingdom and Germany (Table 2). Cardiff University in the United Kingdom had the most extensive foreign cooperation (centrality = 0.12), followed by the National University of Singapore (centrality = 0.11).

**Table 2:** Top 10 institutions with the most publications from 2006 to 2021.

**Institution**	**Country**	**Publication**	**Centrality**
National University of Singapore	Singapore	48	0.11
Sun Yat-Sen University	China	41	0.05
Australian National University	Australia	41	0.07
Capital Medical University	China	34	0.07
Singapore Eye Research Institute	Singapore	32	0.08
University of Melbourne	Australia	31	0.06
University of Houston	USA	29	0.03
Singapore National Eye Center	Singapore	27	0.08
Heidelberg University	Germany	24	0.03
Cardiff University	UK	23	0.12

USA, United States of America; UK, United Kingdom.

### Analysis of authors

**Figure 6:**
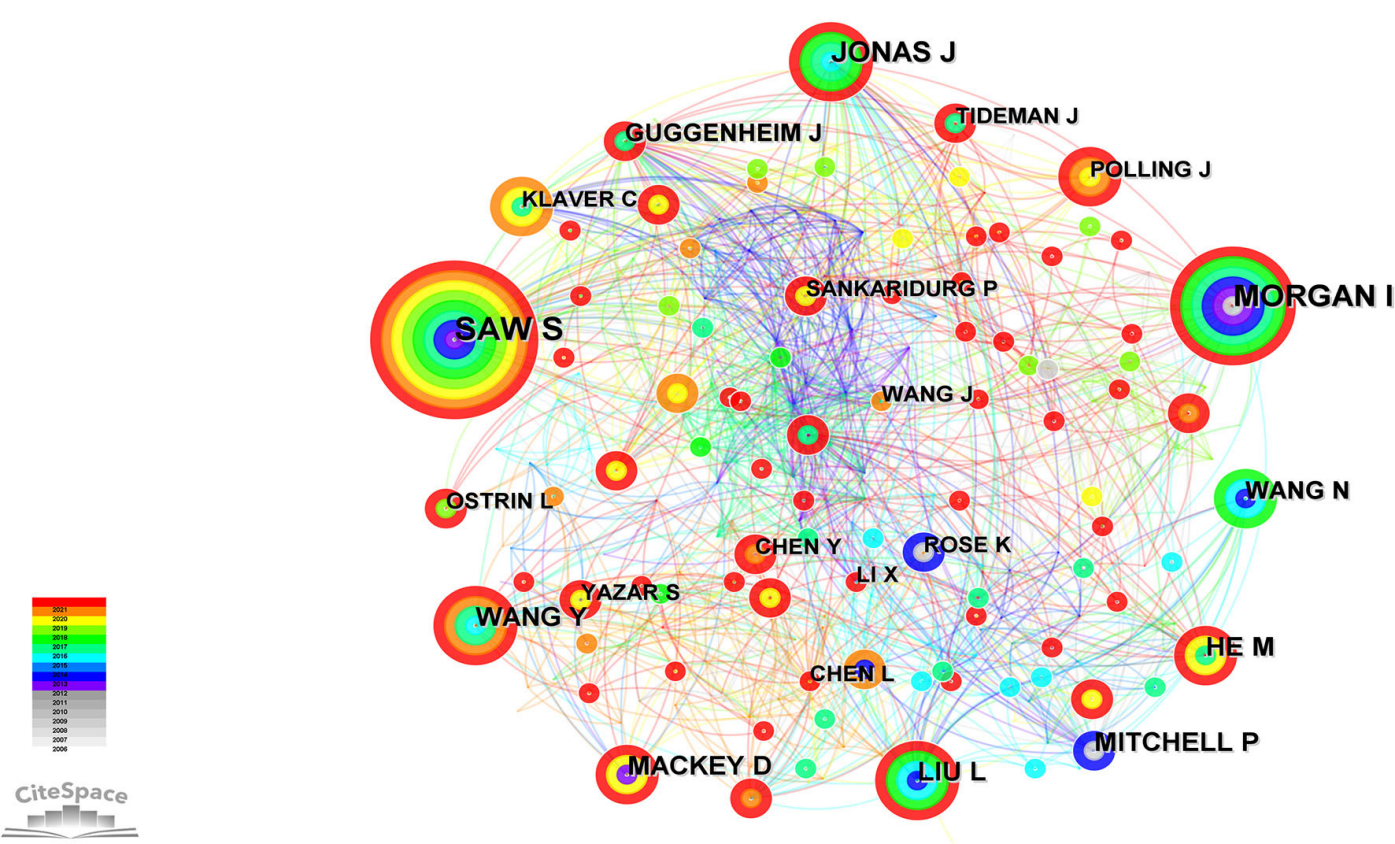
Authors of studies on the relationship between outdoor activities and myopia from 2006 to 2021.

Four hundred and fourteen authors published papers related to this field. The network diagram of the authors' published papers was constructed using CiteSpace, and it consisted of 414 nodes and 1,540 links (Figure 6). The *Q* value and *S* value of the network was 0.6177 and 0.8583, respectively. Saw S had the largest number of publications (*n* = 39). Morgan I (*n* = 27) and Jonas J (*n* = 23) ranked second and third, respectively.

### Analysis of keywords

**Figure 7:**
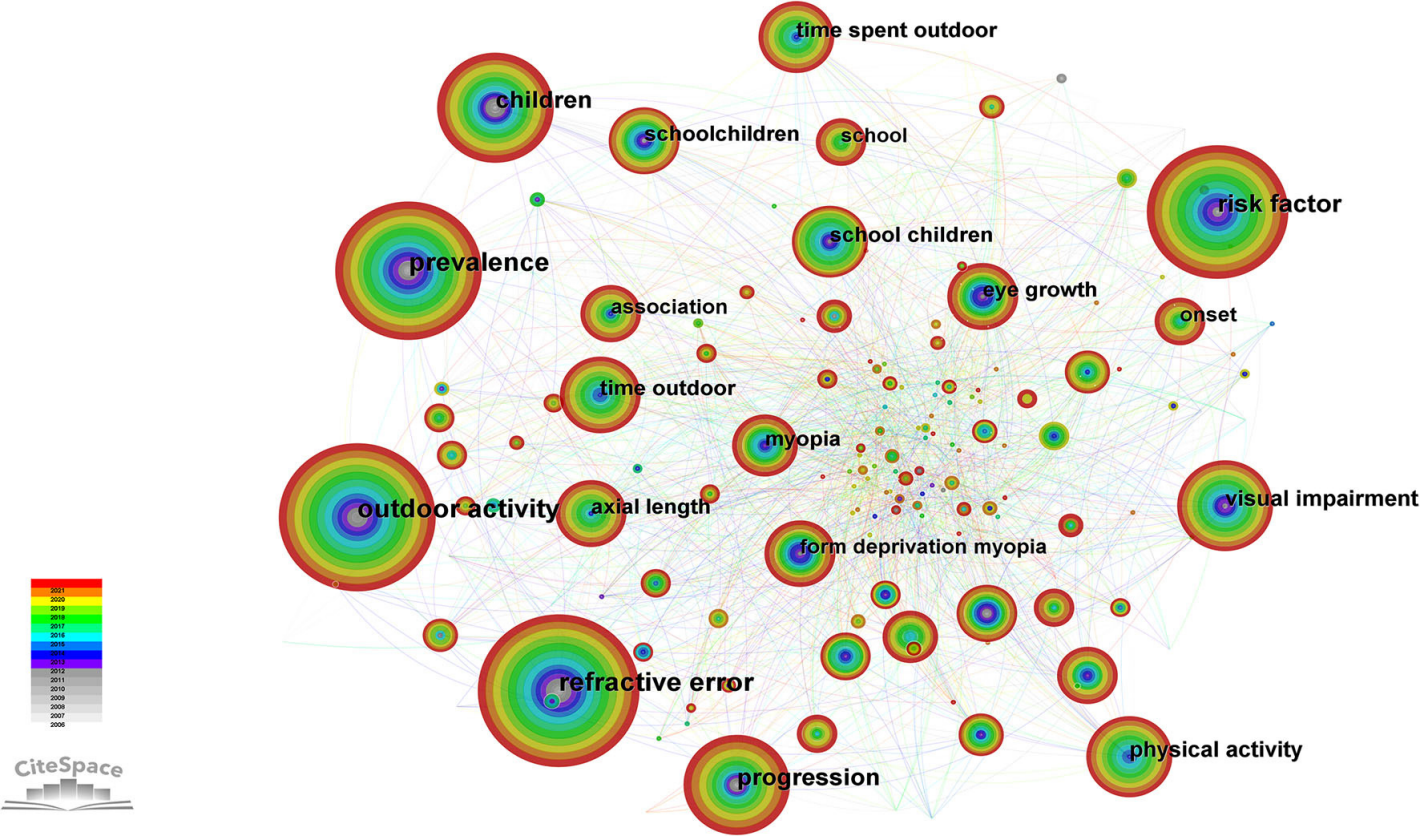
Keywords of studies on the relationship between outdoor activities and myopia from 2006 to 2021.

The 605 publications included in this study comprised 450 keywords, including “refractive error,” “prevalence,” “outdoor activity,” “risk factors,” “progression,” and “children”. The keywords of the network graph were constructed using CiteSpace and consisted of 450 nodes and 1,808 links (Figure 7).

**Figure 8:**
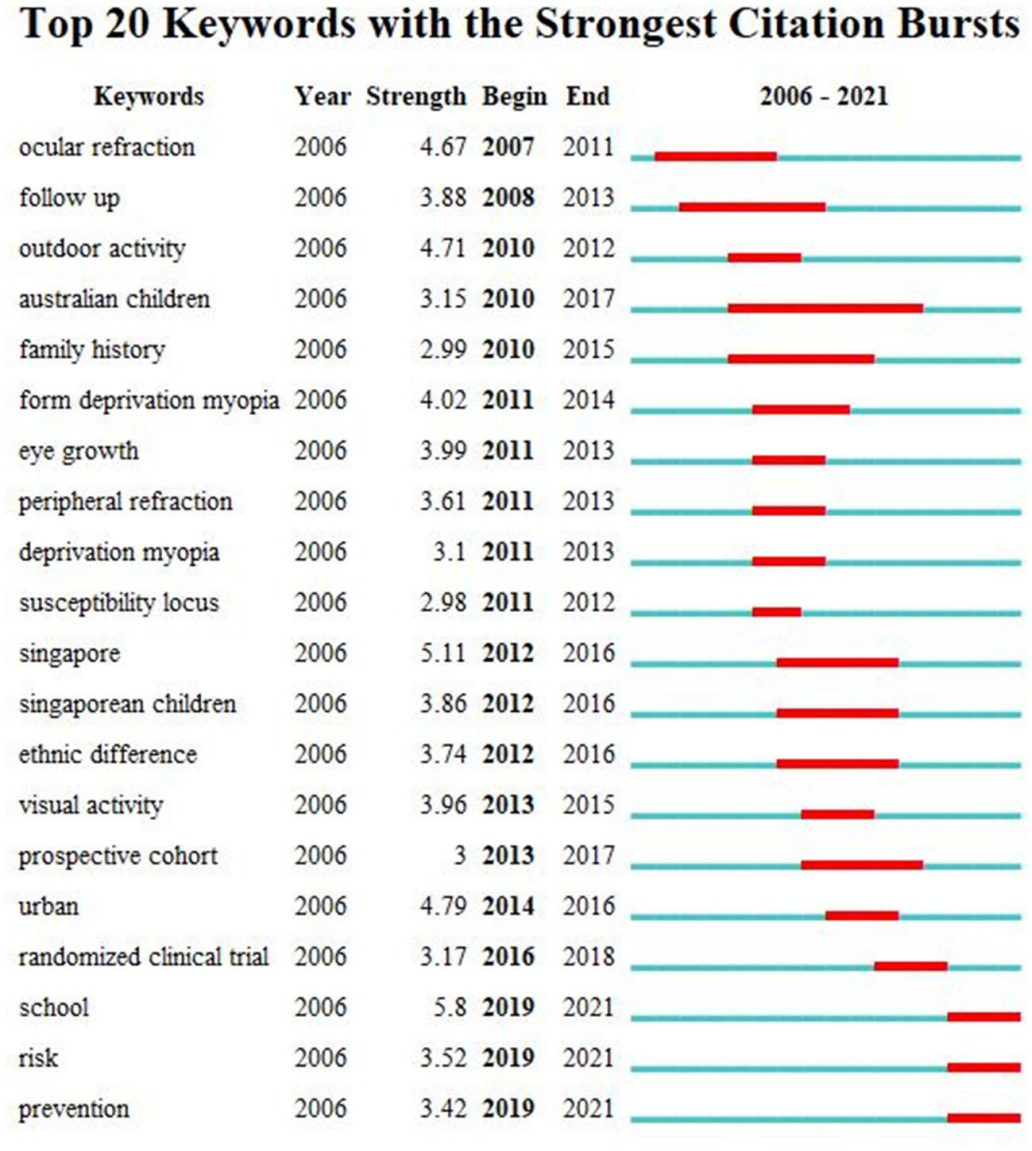
Top 20 keywords with the strongest citation bursts.

We used CiteSpace to conduct a burst detection of keywords in the literature to explore the research frontiers in the relationship between outdoor activities and myopia (Figure 8). “Ocular refraction” and “follow-up” were the first keywords found, between 2007 and 2011 and between 2008 and 2013, respectively. “School,” “risk,” and “prevention” were the latest keywords found since 2019.

### Analysis of cited journals

**Figure 9:**
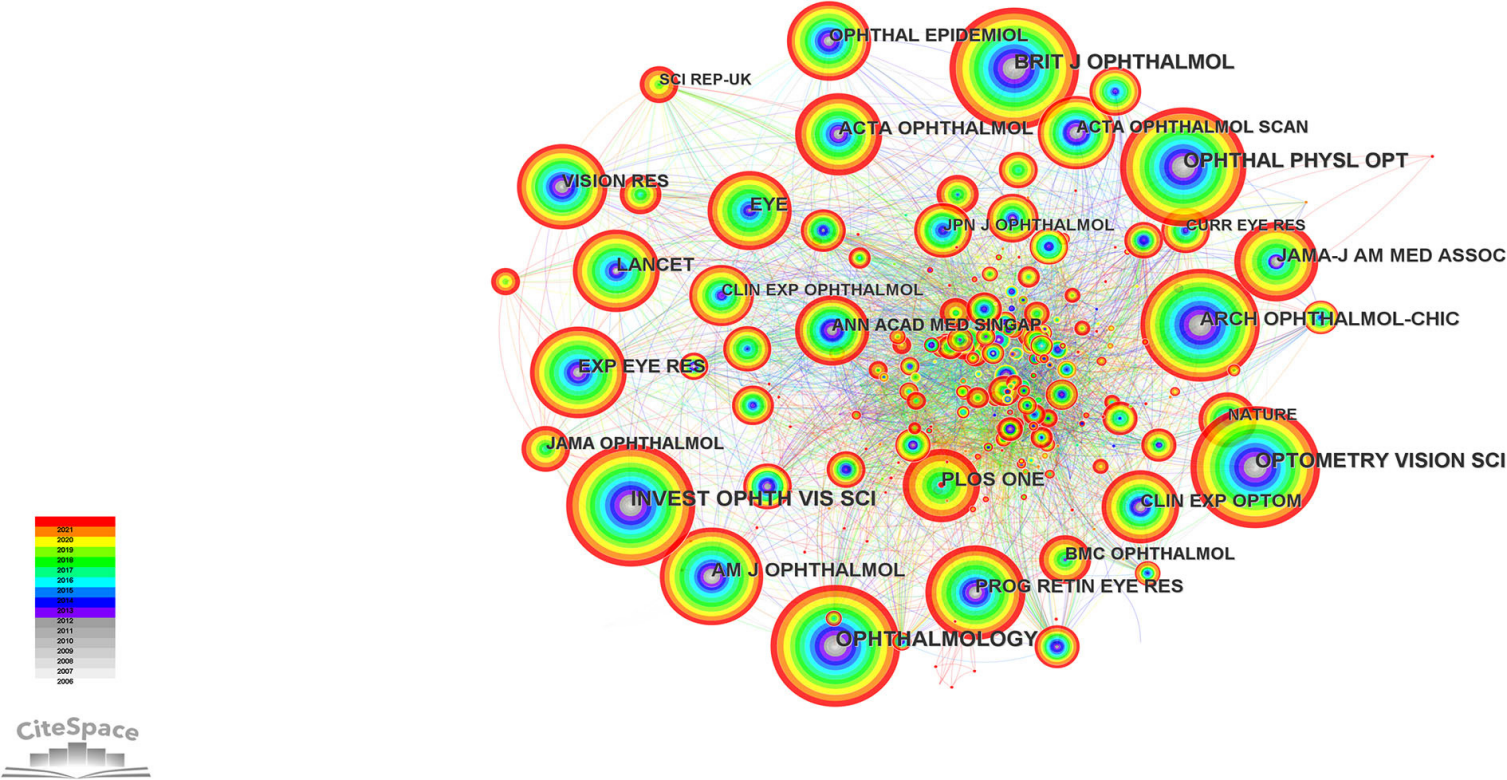
Co-citation analysis of journal on the relationship between outdoor activities and myopia from 2006 to 2021.

Ten journals were cited more than 300 times between 2006 and 2021. The journal co-citation network was constructed using CiteSpace, and it consisted of 668 nodes and 4,596 links (Figure 9). The *Q* value and *S* value of the network was 0.4955 and 0.7582, respectively. As can be seen from the journal co-citation network analysis, the most influential articles on the relationship between outdoor activities and myopia were mainly published in *Investigative ophthalmology* & *visual science, Ophthalmology, Optometry and Vision Science, British Journal of Ophthalmology* and *Ophthalmic and physiological optics* (Table 3). *American journal of ophthalmology* had the biggest centrality (0.03), followed by *Ophthalmology* (0.01), *PLOS ONE* (0.01), *British Journal of Ophthalmology* (0.01) and *Experimental eye research* (0.01). According to Journal Citation Reports in 2021, Five journals ranked Q1. According to impact Factor of journals in 2021, *Progress in Retinal and Eye Research* (19.704) ranked first, followed by *Ophthalmology* (14.277).

**Table 3:** Top 10 Journals with the most cited from 2006 to 2021.

**Journal**	**Subject**	**Cited frequency**	**Centrality**	**Impact factor (2021)**	**Journal citation reports (2021)**
Investigative ophthalmology & visual science	Ophthalmology	592	0	4.925	Q1
Ophthalmology	Ophthalmology	581	0.01	14.277	Q1
Optometry and Vision Science	Ophthalmology	485	0	2.106	Q3
British Journal of Ophthalmology	Ophthalmology	484	0.01	5.908	Q2
Ophthalmic and physiological optics	Ophthalmology	456	0	3.992	Q2
Archives of Ophthalmology	Ophthalmology	382	0	8.253	Q1
PLOS ONE	Multidisciplinary sciences	346	0.01	3.752	Q2
American journal of ophthalmology	Ophthalmology	315	0.03	5.488	Q1
Experimental eye research	Ophthalmology	304	0.01	3.770	Q2
Progress in Retinal and Eye Research	Ophthalmology	301	0	19.704	Q1

Archives of Ophthalmology was renamed as JAMA Ophthalmology in 2013.

### Analysis of cited references

**Figure 10:**
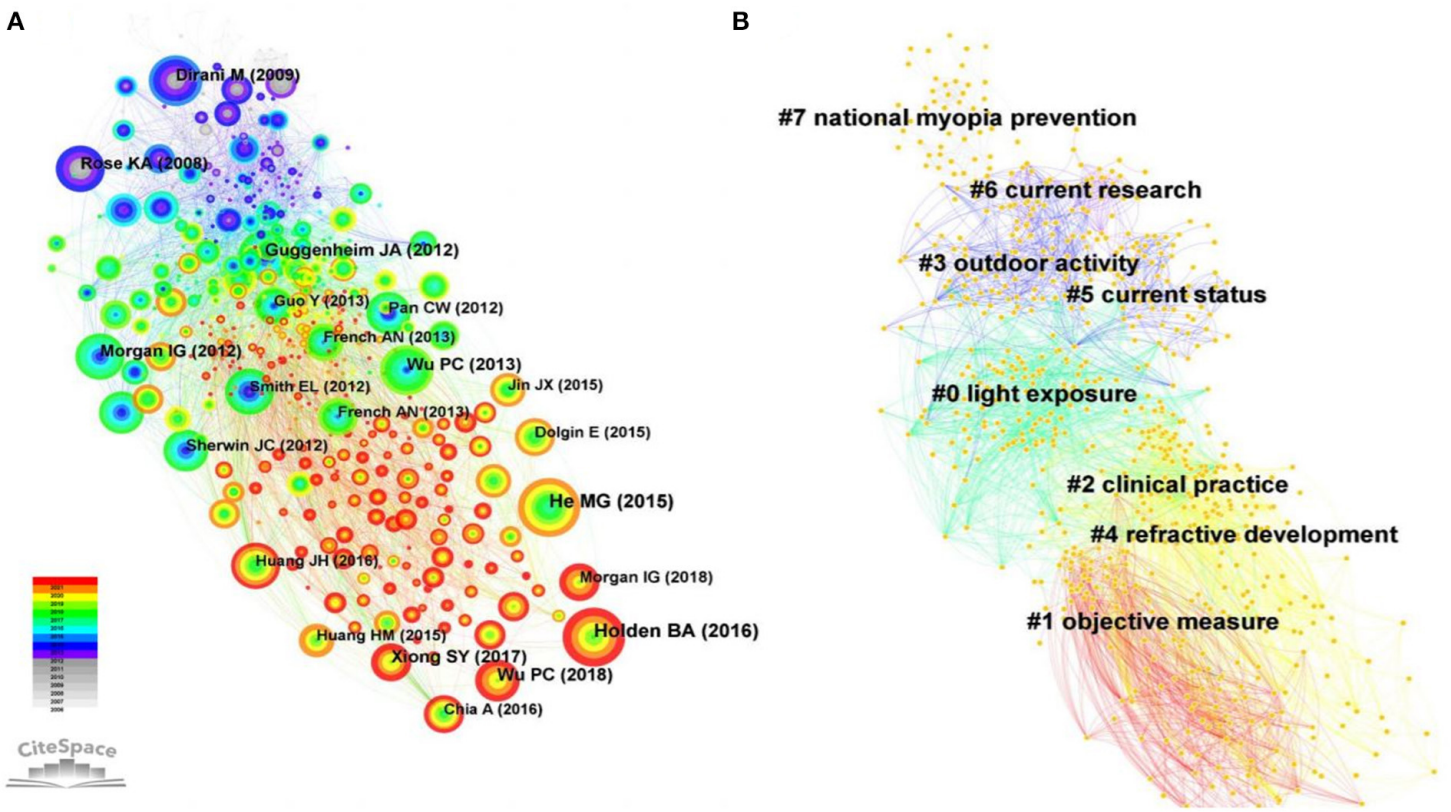
**(A)** Co-citation analysis of references on the relationship between outdoor activities and myopia from 2006 to 2021. **(B)** The clustering of cited references on the relationship between outdoor activities and myopia from 2006 to 2021.

Three publications were cited more than 100 times between 2006 and 2021. “Global Prevalence of Myopia and High Myopia and Temporal Trends from 2000 through 2050” (3), published by Holden B.A. in *Ophthalmology*, was the most cited paper in this field with 177 citations, followed by “Effect of Time Spent Outdoors at School on the Development of Myopia Among Children in China: A Randomized Clinical Trial” (30) with 161 citations and “Myopia Prevention and Outdoor Light Intensity in a School-Based Cluster Randomized Trial” (9) with 102 citations (Figure 10). The *Q* value and *S* value of the reference co-citation network was 0.4025 and 0.767, respectively.

A cluster analysis of the cited references was performed (Figure 10). The citation network of the references was divided into eight citation clusters (*Q* value = 0.6177, *S* value = 0.8583): #0 light exposure, #1 objective measure, #2 clinical practice, #3 outdoor activity, #4 refractive development, #5 current status, #6 current research, and #7 national myopia prevention.

## Discussion

Myopia has become a global public health concern (30). Previous studies have shown that outdoor activity is one of the most important environmental factors for myopia (31), and increasing the time spent outdoors can reduce the risk of myopia (32, 33). Web of Science is a world-renowned retrieval tool for research publications and citations. Users can comprehend the research status and trends in a particular field by statistical analysis of the literature included in this tool. Bibliometrics is a method of indexing and analyzing research directions and hotspots. Conducting bibliometrics through the Web of Science Core Collection database has become popular (34). CiteSpace is the mainstream software used in bibliometric research, which can provide users with a scientific atlas of a certain research field (35). We used CiteSpace to analyze and outline research trends in the relationship between outdoor activities and myopia over the past 15 years.

Studies have shown that myopia can be influenced by innate factors and acquired factors. Epidemiological investigations have shown that myopia is related to outdoor activities (36). The randomized controlled experiment conducted by He M from Sun Yat-Sen University shows that the outdoor exercise group can significantly reduce the prevalence of myopia among adolescents by adding 40 min of outdoor activity per week in school compared with the control group (30). Wu PC from Kaohsiung Chang Gung Memorial Hospital conducted an intervention study among Grade 1 students from 16 schools and found that the students from outdoor exercise intervention group had significantly reduced axial elongation and the risk of myopia compared with the control group (9). The number of publications was divided into two time periods in the present study, with the year 2010 set as the boundary. Only a few related studies were published before 2010, with the number of publications and citations being less than 10 and 100, respectively. This showed that the relationship between outdoor activities and myopia did not attract widespread attention in the past. However, an opposite trend was observed after 2010, with the number of research articles analyzing the association between outdoor activities and myopia gradually increasing. Saw S from National University of Singapore had the largest number of publications. *Investigative ophthalmology* & *visual science* is the most important journal, followed by *Ophthalmology* and *Optometry and Vision Science*.

China, the United States of America, Australia, and Singapore were the primary countries to conduct research on the association between outdoor activities and myopia in recent years. China was the only developing country among the top 10 countries with the largest number of publications. Nevertheless, it contributed the largest number of publications in this field. The number of publications in China has increased rapidly, especially after 2012, but research cooperation between China and other countries needs to be strengthened. It can be seen that the United States of America played a leading role in this field, followed by Australia. In recent years, the association between outdoor activities and myopia had attracted the attention of scholars all around the world. This trend could be attributed to the increasing prevalence of myopia worldwide. The prevalence of myopia in East Asian countries, such as Singapore and China, is significantly high (37), and the increasing prevalence of myopia in China has attracted considerable attention from researchers and the government (38).

Keywords reflect the theme of an article. High-frequency keywords can be regarded as hot topics in related research fields. Frontiers in research can be recognized by detecting keywords with rapid growth in frequency (39). Analysis of burst keywords showed that students were the main focus of researchers, while the risk and preventive measures for myopia were the hotspots for future studies. These findings suggest that studies on the association between outdoor activities and myopia among students should be strengthened.

It was observed that outdoor activities could reduce the risk of myopia. However, further studies are required to determine differences in their protective effects on children and adolescents based on region, gender, and ethnicity (30, 40–43). Additionally, although several studies have reported that outdoor activities can reduce the prevalence of myopia (44–46), there is insufficient evidence showing that outdoor activities can inhibit its progression (47). Therefore, further researches are needed to determine the pathophysiology of outdoor activities in inhibition of progression of myopia. We primarily used a survey questionnaire to determine the importance of outdoor activity duration. Likewise, few randomized clinical trials and longitudinal follow-up studies have explored the effects of outdoor activities on myopia. There is a lack of consensus regarding the intensity or duration of outdoor activities most suitable to prevent myopia.

This was the first analysis using bibliometrics and visualization techniques to explore the impact of outdoor activities on myopia by scrutinizing published researches between 2006 and 2021. The *Q* value and *S* value of network diagram in the present study was greater than 0.3 and greater than 0.5, respectively, which indicates that the community structure of the network of scientific knowledge is significant and the clustering of the network of scientific knowledge is reasonable. Through this study, we identified the hotspots along with renowned research institutions and teams in this field. We also understood the ideas and directions of future research. However, the present study had some limitations. First, the language of included publications was limited to English, which resulted in a bias in literature selection. Second, the articles were retrieved using subject terms, and thus some relevant publications may have been omitted from this study. Third, CiteSpace was developed based on the data format of the Web of Science database, and data from the Web of Science database can be directly imported into CiteSpace for visual analysis (48). It is therefore that we only retrieved the literature in the Web of Science database as the analysis object in this study, and did not include other publications not included in Web of Science database. In fact, CiteSpace provides format conversion for literature from other databases such as Scopus, so the literature from other databases can also be imported into CiteSpace for analysis after format conversion (48). Fourth, CiteSpace cannot analyze and evaluate the quality of publications. Although this study has some limitations, it still reveals the trends and hotspots of future research in this field.

In conclusion, we analyzed literature on the relationship between outdoor activities and myopia, using the Web of Science database. The impact of outdoor activities on myopia has attracted the attention of researchers, resulting in an annual increase in the number of publications. China was the main contributor of studies in this field. The breadth and depth of cooperation between countries or institutions must be strengthened to analyze this relationship. The National University of Singapore, Saw S and *Investigative ophthalmology* & *visual science* were the most influential institution, author and journal, respectively. Moreover, additional evidence is required to explore the effects of outdoor activities on myopia. Likewise, more research is needed to improve our understanding of the association between outdoor activities and myopia and reduce the risk of myopia among school students. In general, researchers can benefit from the bibliometric analysis in this study, because they can understand the knowledge structure, hotspots and frontiers of this field.

## Data availability statement

The original contributions presented in the study are included in the article/supplementary material, further inquiries can be directed to the corresponding author.

## Author contributions

JM,HZ, DZ, JF, and MJ: contributed equally. JM, HZ, DZ, JF, MJ, ML, XS, YC, and SZ: conceptualization and methodology. HZ, DZ, JF, MJ, XS, and YC: data curation. JM, HZ, DZ, JF, MJ, and SZ: formal analysis. SZ: supervision. JM: visualization and writing (original draft). HZ, DZ, JF, MJ, and SZ: writing (review and editing). All authors have approved the final version of the manuscript.

## Funding

This work was supported by Sanming Project of Medicine in Shenzhen (No. SZSM202011015) and Natural Science Foundation of Guangdong Province (No. 2021A1515011090).

## Conflict of interest

The authors declare that the research was conducted in the absence of any commercial or financial relationships that could be construed as a potential conflict of interest.

## Publisher's note

All claims expressed in this article are solely those of the authors and do not necessarily represent those of their affiliated organizations, or those of the publisher, the editors and the reviewers. Any product that may be evaluated in this article, or claim that may be made by its manufacturer, is not guaranteed or endorsed by the publisher.
